# Cytotoxic Effects of Betel Quid and Areca Nut Aqueous Extracts on Mouse Fibroblast, Human Mouth-Ordinary-Epithelium 1 and Human Oral Squamous Cell Carcinoma Cell Lines

**DOI:** 10.31557/APJCP.2020.21.4.1005

**Published:** 2020-04

**Authors:** Badr Abdullah Al-Tayar, Azlina Ahmad, Mohamad Ezany Yusoff, Siti Fadilah Abdullah, Noor Khairiena Mohamad, Siti Nurnasihah Md Hashim, Shosei Kishida, Michiko Kishida, Norifumi Nakamura, Toshiro Kibe, Masitah Hayati Harun

**Affiliations:** 1 *School of Dental Sciences, Universiti Sains Malaysia, Health Campus, 16150 Kubang Kerian, Kelantan, Malaysia, *; 2 *Graduate School of Medical and Dental Sciences, Kagoshima University, 8-35-1 Sakuragaoka, Kagoshima, Japan. *

**Keywords:** Areca nut, betel quid, cell lines, cytotoxicity, oral cancer

## Abstract

**Background:**

Betel quid chewing is more common among the older generation in rural areas of Malaysia. Oral cancer in Asia has been associated with the habit of chewing betel quid and areca nut.

**Objective:**

This study aims to investigate the cytotoxic effects of betel quid and areca nut extracts on the fibroblast (L929), mouth-ordinary-epithelium 1 (MOE1) and oral squamous cell carcinoma (HSC-2) cell lines.

**Methods:**

L929, MOE1 and HSC-2 cells were treated with 0.1, 0.2 and 0.4 g/ml of betel quid and areca nut extracts for 24, 48 and 72 h. MTT assay was performed to assess the cell viability.

**Results:**

Both extracts, regardless of concentration, significantly reduced the cell viability of L929 compared with the control (P<0.05). Cell viability of MOE1 was significantly enhanced by all betel quid concentrations compared with the control (P<0.05). By contrast, 0.4 g/ml of areca nut extract significantly reduced the cell viability of MOE1 at 48 and 72 h of incubation. Cell viability of HSC-2 was significantly lowered by all areca nut extracts, but 0.4 g/ml of betel quid significantly increased the cell viability of HSC-2 (P<0.05).

**Conclusion:**

Areca nut extract is cytotoxic to L929 and HSC-2, whereas the lower concentrations of areca nut extract significantly increased the cell viability of MOE1 compared to the higher concentration and control group. Although betel quid extract is cytotoxic to L929, the same effect is not observed in MOE1 and HSC-2 cell lines. Further investigations are needed to clarify the mechanism of action.

## Introduction

Chewing betel quid is one of the traditional but harmful habits in a number of Southeast Asian nations. This practice has been reported to reduce stress, increase the alertness and enhance the psychological well-being of chewers (Winstock, 2002; Sharan and Choudhury, 2010). Approximately 10.7%–43.6% of men and 1.8%–34.9% of women in Taiwan, Mainland China, Nepal and Sri Lanka chew betel quid (Lee et al., 2011). In Malaysia, this habit is more prevalent among the older generation in rural areas, with a higher prevalence in women (29.5%) than in men (9.8%) (Ghani et al., 2011; Lee et al., 2011). Oral cancer in some parts of Asia and the Pacific has been associated with chewing betel quid (Gupta and Johnson, 2014). In Malaysia, the higher cancer risk noted in the Indian ethnic group and the indigenous people of Sabah and Sarawak compared with ethnic groups may be attributed to the habit of chewing betel quid (Zain et al., 1997; Zain and Ghazali, 2001; Ghani et al., 2011). A retrospective study involving 118 Malay oral cancer patients in the Hospital Universiti Sains Malaysia reported that 22.9% of the patients chewed betel quid (Razak et al., 2009).

Betel quid generally consists of areca nut, betel leaf, lime and other possible constituents, such as tobacco and essences, although the actual composition varies depending on the region (Sharan et al., 2012; Chen et al., 2015). Chin and Lee (1970) studied 212 Indian and 48 Malay betel quid chewers in Malaysia. In this study, the betel quid consumed by 167 Indian subjects consisted of betel leaf, slaked lime, tobacco and powdered or sliced dried areca nut while the rest of the Indian subjects chewed betel quid without tobacco. In contrast, 45 Malays chewed betel quid, comprising betel leaf, gambir, slaked stone lime and areca nut, without tobacco. A number of, 39 chewers consumed betel quid without gambir. The main component of betel quid is the Areca catechu seeds, commonly known as areca nut. The leaf of Piper betel is usually chewed along with areca nut. In addition to these two plant materials, slaked lime, which is quarried from limestone, is often concocted with the quid mixture. Another ingredient that may be added to the quid mixture is gambir, which is an aqueous extract from the leaves and young shoots of Uncaria gambier, a climbing shrub indigenous to the Malay Archipelago. 

Areca nut and betel quid with or without tobacco have been classified as group 1 human carcinogens (Pankaj, 2010). Areca nut contains tannin and areca alkaloids, such as arecoline and arecaidine, which are involved in carcinogenesis (Sharan and Choudhury, 2010). 

Areca nut-specific nitrosamines, which are mutagenic, genotoxic and tumourigenic in experimental animals, are produced by the nitrosation of areca nut alkaloid (Jeng et al., 2001). Instead of crude betel quid extract, single chemical elements, such as arecoline bromide, have been applied in previous studies (Chiang et al., 2007; Lin et al., 2011; Liu et al., 2016). Crude plant extracts reportedly exhibit a more remarkable *in vitro* effect compared with isolated constituents at the same dosage (Rasoanaivo et al., 2011). To date, few experimental studies on Malaysian betel quid preparations have been conducted. In the current study, we investigated the cytotoxicity of crude betel quid and areca nut aqueous extracts on mouse fibroblast (L929), human mouth-ordinary-epithelium 1 (MOE1) and human oral squamous cell carcinoma (HSC-2) cell lines.

## Materials and Methods

*Preparation of areca nut and betel quid extracts*

Dried areca nut flakes and betel quid were purchased from a source in Kelantan, Malaysia. The betel quid consisted of P. betel leaf, slaked lime and dried areca nut flakes. Aqueous extracts were prepared as previously described by Sazwi et al. ,(2013) with some modifications. Specific weights of areca nut and betel quid were separately homogenised with deionised water in a blender for 3 min and soaked for 6 h. The mixture was centrifuged at 2,500 rpm for 5 min to remove the insoluble material. The supernatant was filtered using Whatman filter paper no.1 (GE Healthcare, New Jersey, USA) before the extracts were concentrated using a rotary evaporator. The concentrated extracts were freeze-dried and stored at -20°C until further use. 

*Preparation of stock and working concentrations of betel quid and areca nut*

Freeze-dried areca nut (4 g) and betel quid powder (4 g) were diluted in 10 ml of complete media to produce the main stocks (0.4 g/ml) of areca nut and betel quid. The stocks were placed in a shaker incubator for 24 h and filtered using a nylon syringe filter (0.22 µm). The working concentrations were prepared by serially diluting the main stock with media. Three different concentrations (0.1, 0.2, and 0.4 g/ml) were prepared for each stock. 

*Culture of L292, MOE1 and HSC-2*

The L929 cell line was purchased from the American Type Culture Collection (ATCC, Manassas, VA, USA) and grown in Dulbecco’s modified Eagle’s medium (DMEM) supplemented with 10% foetal bovine serum (FBS; Gibco, Grand Island, NY, USA). The MOE1 cell line was provided by Dr. Shosei Kishida (Department of Biochemistry and Genetics, Kagoshima University, Japan) and cultured in defined keratinocyte serum-free medium (defined K-SFM) supplemented with 1 ml of defined K-SFM growth supplement and 1% penicillin G-streptomycin (Gibco, Grand Island, NY, USA) (Kibe et al., 2011). HSC-2 cell line was purchased from Riken Cell Bank (Tsukuba, Japan) and cultured in minimum essential medium (MEM) supplemented with 10% FBS (Gibco, Grand Island, NY, USA). All cell lines were incubated under a humidified 5% CO_2_ atmosphere at 37^o^C. 

*Cytotoxicity assay*

3-(4,5-Dimethyl thiazol-2yl)-2, 5-diphenyltetrazolium bromide (MTT) (Invitrogen Eugene, OR, USA) was used to determine the cytotoxicity of the extracts on the L929, MOE1 and HSC-2 cell lines according to Mosmann (1983). L929, MOE1 and HSC-2 cells were seeded separately at 3×104 cells/ml in sterile 24-well microliter plates and incubated at 37 °C under a humidiﬁed atmosphere of 5% CO_2_. 

After 24 h, the culture media were discarded from each well and replaced with 1 ml of extracts at different concentrations (0.1, 0.2 and 0.4 g/ml). The cells were further incubated for 24, 48 and 72 h. Cells in 1 ml of culture medium served as negative controls. 

After treatment, 100 µl of MTT solution was added to each well. After incubation for 4 h, the MTT solution and medium were discarded, and 1 ml of dimethyl sulphoxide (Gibco, Grand Island, NY, USA) was added to each well. The plates were shaken at 300 rpm for 15 min. Then, 100 µl of the solution was transferred to each well in a 96-well plate. The optical densities (ODs) of the dissolved formazan were read at 570 nm by using a microplate spectrophotometer (Tecan, Männedorf, Switzerland). The viability ratio (%) at each dilution was determined using the following formula: (Mean OD of treated cells/Mean OD of control cells) × 100.

*Statistical analysis*

Data entry and analysis were performed using SPSS (version 22.0; IBM, Chicago, USA). Experiments were repeated at least thrice, and the data are presented as means±standard error. The results were analysed using one-way ANOVA with Scheffe and Games–Howell post–hoc test and Kruskal Wallis complemented by Mann Whitney U-test to compare the means at p<0.05.

## Results

*Cytotoxicity of betel quid and areca nut extracts on L929*

**Figure 1:**
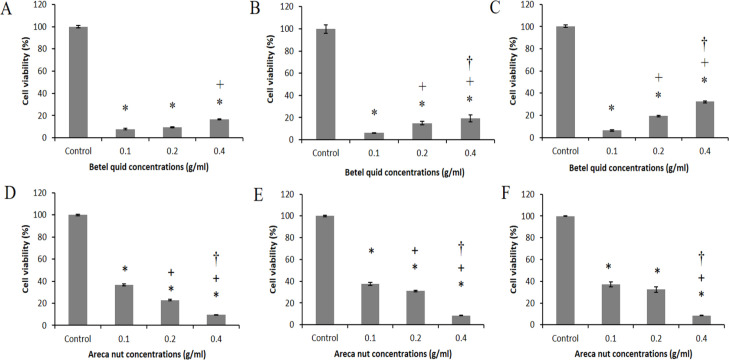
Cell Viability of L929 Treated with Betel Quid and Areca Nut Extracts, respectively. (A and D) 24h, (B and E) 48 h, and (C and F) 72h.*Significant with control, + Significant with 0.1g/ml, and † Significant with 0.2 g/ml (*P*<0.05).

Figure 1 shows the cytotoxic effects of the prepared extracts on L929 cell line. Both extracts at all concentrations significantly resulted in cell death compared with the control group (p<0.05). Treatment with both extracts for all concentrations decreased cell viability to below 40%. Cell viability was also significantly higher for the highest betel quid extract concentration (0.4 g/ml) compared with other concentrations at 48 and 72 h of incubation (p<0.05). In contrast, areca nut at 0.4 g/ml significantly reduced the cell viability compared with the lower concentrations after 24, 48 and 72 h (p<0.05).

*Cytotoxicity of betel quid and areca nut extracts on MOE1*

**Figure 2:**
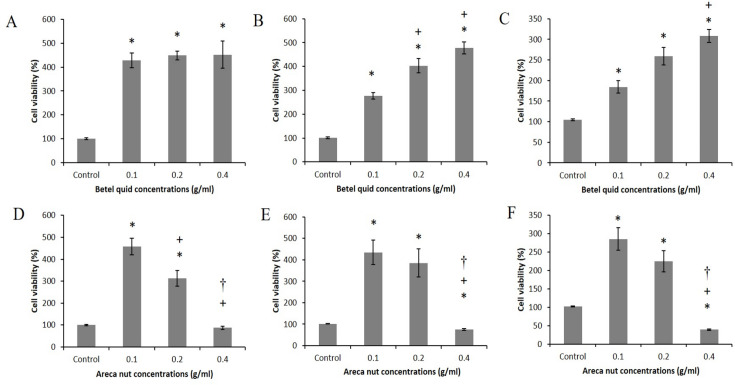
Cell Viability of MOE1 Treated with Betel Quid and Areca Nut Extracts, respectively. (A and D) 24, (B and E) 48, and (C and F) 72h. * Significant with control, + Significant with 0.1g/ml, and † Significant with 0.2 g/ml (*P*<0.05).

Figure 2 illustrates the cytotoxic effects of betel quid and areca nut extracts on the MOE1 cell line. Areca nut extracts at 0.1 and 0.2 g/ml significantly increased cell proliferation compared with the control group after 24, 48 and 72 h of incubation (p<0.05). In contrast, the highest concentration of areca nut (0.4 g/ml) significantly decreased cell viability compared with the control group after 48 and 72 h of incubation (p<0.05). All betel quid concentrations significantly increased the cell viability of MOE1 compared with the control group after 24, 48 and 72 h of incubation (p<0.05).

*Cytotoxicity of betel quid and areca nut extracts on HSC-2*

**Figure 3:**
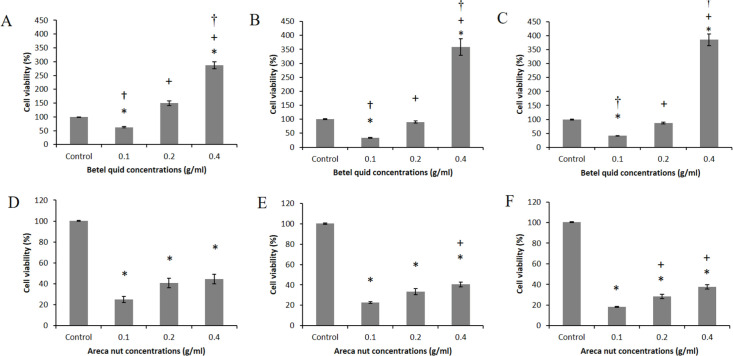
Cell Viability of HSC-2 Treated with Betel Quid and Areca Nut Extracts, respectively. (A and D) 24, (B and E) 48, and (C and F) 72h. *Significant with control, + Significant with 0.1g/ml, and †Significant with 0.2 g/ml (P<0.05)

Figure 3 displays the cytotoxic effect of the extracts on the HSC-2 cell line. All areca nut extracts significantly reduced the cell viability compared with the control group, but the betel quid (0.4 g/ml) significantly increased the cell viability of HSC-2 compared with the control and the lower concentrations after 24, 48 and 72 h of incubation (p<0.05).

## Discussion

Population-based studies indicated that the risk of oral cancer increases by chewing areca nut and betel quid (Warnakulasuriya et al., 2002; Salian et al., 2016). This habit can also adversely affect the fibrous connective tissues, resulting in oral submucous fibrosis, which is a potentially malignant disorder of the oral mucosa (Prabhu et al., 2014). Additionally, long-term areca nut chewing has been associated with detrimental effects on the periodontium and temporomandibular joint (TMJ) because of the excessive masticatory forces transmitted to the TMJ (Hsiao et al., 2015; Nawaz, 2015). 

In the present study, the cytotoxic effect of the areca nut was indicated by the significantly lowered cell viability of L929 with increasing concentration of the areca nut extract. This result is consistent with the findings of a previous study that reported the cytotoxic effect of areca nut extract on fibroblasts (Yeh et al., 2016). Areca nut contains arecoline, which is a cytotoxic agent to many cell lines, such as gingival fibroblasts, cementoblasts and endothelial cells (Chang et al., 2001; Ullah et al., 2014; Chen et al., 2015). The toxicity of the chemicals may lead to DNA damage in the exposed cells, thereby leading to carcinogenesis (Bogdanffy and Valentine, 2003). Therefore, the progression of oral cancer may be aggravated by the cytotoxicity of areca nut. 

Although the cell viability of L929 was significantly reduced by all betel quid extracts, betel quid at a high concentration of 0.4 g/ml increased the cell viability compared with the lower concentrations of this extract. This result suggests that the betel quid extract may be a factor in the alteration of cell cycle regulation. Lin et al., (2003) reported an increase in the percentage of cells distributed after treatment with betel quid (areca nut, Piper linn flower and red lime) in the synthesis phase, thereby indicating the elevated DNA synthesis of the cells. This result may be attributed to the generation of toxic species, such as reactive oxygen species and other potential tumour promoters, during the interaction among betel quid components. Hence, combining multiple compounds in an extract results in diverse cellular effects compared with a single-compound extract (Rasoanaivo et al., 2011). 

Sari et al., (2017) reported that all areca nut extract concentrations (160-2,560 µg/ml) in their study increased the proliferation of human keratinocyte (HaCat) cell line by more than 100% compared with the control group. In the current study, the proliferation of MOE1 was significantly increased by areca nut extract at lower concentrations. In addition, areca nut extract at the highest concentration had cytotoxic effect on MOE1 cells. These contrasting observations may be due to the difference in areca nut extract dosages. 

The proliferation of MOE1 was significantly enhanced by the betel quid extract with the highest concentration. Faouzi et al., (2018) investigated the role of the extracts in the production of inflammatory cytokines such as interleukin-8 (IL-8) from some immune cells, thereby enhancing cell proliferation. IL-8, which is important in cancer growth, is secreted by oral squamous cancer cells and also used as the biomarker for this malignancy (Watanabe et al., 2002; Sahibzada et al., 2017). 

The results of the current study are consistent with Sari et al., (2017), who reported the cytotoxic effect of areca nut extract on HSC-2 cells. However, the present results indicated that the highest concentration of betel quid increased the proliferation of the HSC-2 cells. This effect was not observed in the MOE1 and HSC-2 cell lines after treatment with areca nut. Although only the cell viability of selected cell lines was measured in the present study, the exact mechanism underlying the proliferation and cytotoxicity of both extracts is currently being investigated. 
